# The real-world anti-inflammatory effect of SGLT2i in patients with chronic heart failure

**DOI:** 10.25122/jml-2025-0011

**Published:** 2025-02

**Authors:** Alexandru Mircea Arvunescu, Ruxandra Florentina Ionescu, Silviu Ionel Dumitrescu, Ondin Zaharia, Ioan Tiberiu Nanea

**Affiliations:** 1 Department of Cardio-Thoracic Pathology, Faculty of Medicine, Carol Davila University of Medicine and Pharmacy, Bucharest, Romania; 2 Department of Internal Medicine and Cardiology, Prof. Dr. Th. Burghele Clinical Hospital, Bucharest, Romania; 3 Department of Cardiology I, Dr. Carol Davila Central Military Emergency Hospital, Bucharest, Romania; 4 Department of Cardiology, Titu Maiorescu University, Faculty of Medicine, Bucharest, Romania

**Keywords:** heart failure, chronic inflammation, left ventricular ejection fraction, SGLT2i, HFrEF, HFpEF, fibrinogen, C-reactive protein, ESR, CHF, Chronic Heart Failure, CRP, C-Reactive Protein, EF, Ejection Fraction, ESC, European Society of Cardiology, HF, Heart Failure, HFmrEF, Heart Failure With Mildly Reduced Ejection Fraction, HFpEF, Heart Failure With Preserved Ejection Fraction, HFrEF, Heart Failure With Reduced Ejection Fraction, IL-6, Interleukin 6, LVEF, Left Ventricular Ejection Fraction, NT-proBNP, Amino-Terminal End of Pro Brain Natriuretic Peptide, OGMT, Optimally Tolerated Guideline-Directed Medical Treatment, SGLT2i, Sodium-Glucose Co-Transporter 2 Inhibitors, TNF, Tumor Necrosis Factor

## Abstract

Inflammation plays a major role in the etiology of chronic heart failure and in inducing the progression to end-stage heart failure. This chronic inflammation, which accompanies heart failure, is not only local but also systemic and is usually in a state of low-grade but constant activation. Because there is an interrelation between systemic inflammation and neurohormonal activation, almost all anti-remodeling classes of medication have been evaluated for a potential and hidden anti-inflammatory effect. This study aimed to evaluate the effect of sodium-glucose co-trans-porter 2 inhibitors (SGLT2i) (Dapagliflozin or Empagliflozin) on inflammation measured by C-reactive protein levels, erythrocyte sedimentation rate (ESR) and fibrinogen in patients with chronic heart failure when administered together with other standard heart failure medications. We retrospectively enrolled 220 patients with chronic heart failure admitted to our hospital from January 2021 until March 2023. The study included two visits, T0 (the initial visit) and T1 (after six months), to assess if SGLT2i initiation after the first visit (T0) had an effect on the levels of inflammatory biomarkers. SGLT2i showed a reduction in fibrinogen levels, an effect that was present both in heart failure with reduced ejection fraction (HFrEF) and heart failure with preserved ejection fraction (HFpEF) phenotypes. This was opposite to the dynamics of inflammatory markers in patients who did not receive SGLT2i, where the fibrinogen levels increased in HFrEF and HFpEF subgroups. SGLT2i proved an anti-inflammatory effect, showing a statistically significant reduction in fibrinogen levels in chronic heart failure, irrespective of the phenotype.

## INTRODUCTION

Heart failure (HF) is a very common pathology that produces a great burden of morbidity and mortality around the globe [1]. Although the in-hospital mortality rate ranges between 2% and 17% at first admission, the five-year survival rate after diagnosis is less than 50% [2]. Whether HF affects the ventricular filling during diastole or the ejection of blood during systole, the consequences are clinical symptoms and signs of pulmonary and/or systemic congestion [3]. Chronic heart failure (CHF) is the endpoint of structural heart diseases but also extracardiac pathologies, and its incidence and mortality rate exceed those of many malignant diseases [2].

In order to better understand the underlying mechanisms of this complex syndrome and identify appropriate treatments, CHF has been subdivided into three phenotypes: heart failure with reduced ejection fraction (HFrEF), heart failure with mildly reduced ejection fraction (HFmrEF), and heart failure with preserved ejection fraction (HFpEF) [1]. Among these, HFmrEF is the most recently defined category with the narrowest ejection fraction range. Recent trials suggest that patients with HFmrEF may benefit from the same classes of medications recommended for HFrEF [4].

Systemic inflammation plays a role in the development and progression of CHF in all three phenotypes, and evidence shows a significant correlation between proinflammatory cytokines and adverse cardiovascular events [5].

The systemic echo of HF can be measured through non-specific inflammatory biomarkers and specific proinflammatory cytokines [6]. However, the analysis of these markers shows lower levels in the case of HF than those observed in autoimmune disorders or acute and chronic infections [7].

Whether this chronic low-grade inflammation is the potential cause inducing HF or the proinflammatory cytokines appear as a consequence of HF functional status decline is still an ongoing debate [8].

Although all three HF phenotypes are associated with chronic systemic inflammation, studies show a more significant link between proinflammatory biomarkers and HFpEF [9]. Two other studies (BIOSTAT and COACH) explain this correlation, showing that patients with chronic diseases are exposed to chronic inflammation burden [10].

Furthermore, this low-grade chronic inflammation exerts a cytotoxic effect on the cardiomyocyte cellular structure and disrupts the inner metabolic homeostasis. This destructive process, fueled by oxidative stress, is induced by reactive oxygen species, leading to progressive impairment of systolic and/or diastolic function [11].

In the case of ischemic HFrEF, systemic inflammation can be an effect of the direct myocyte lesion, which appears in the case of prolonged or severe ischemia, the myocardium being infiltrated with neutrophils and monocytes [11]. Chronic inflammation and cardiomyocyte injury are further sustained by the activation of neurohormonal systems (sympathetic nervous system, renin-angiotensin-aldosterone system). During the reparative phase, myofibroblasts synthesize collagen; however, the continued activation of these neurohormonal systems perpetuates inflammation and further myocardial injury.

Sodium-glucose co-transporter 2 inhibitors (SGLT2i) represent a new class of medications with a glycosuric effect used in treating type 2 diabetes mellitus. Clinical trials evaluating their safety have ruled out serious adverse cardiovascular effects and demonstrated significant cardioprotective benefits [12].

These studies were followed by specifically designed studies that evaluated the potential role of SGLT2i in patients with CHF [13]. The results proved statistically significant results in reducing mortality and the rehospitalization risk for patients diagnosed with CHF. These results were consistent throughout the whole class of SGLT2i, imposing them as the first class of medication recommended in CHF across the whole spectrum of left ventricular ejection fraction (LVEF) [14].

Some physiopathological strings that generate these cardioprotective effects are described theoretically, but not all are demonstrated or tested yet [15]. One mechanism is the anti-inflammatory action of SGLT2i, which, as shown in animal studies, reduces oxidative stress and enhances recovery after ischemia-reperfusion injury [16]. Furthermore, a meta-analysis by Theofilis *et al*. revealed that rodents receiving SGLT2i exhibited lower levels of interleukin-6 (IL-6), C-reactive protein (CRP), and tumor necrosis factor-alpha (TNF-α) compared to controls [17].

The objective of this study was to evaluate the effect of SGLT2i on the low-grade chronic inflammation present in CHF, irrespective of LVEF. For this purpose, we compared the blood levels of three common non-specific inflammatory markers— CRP, erythrocyte sedimentation rate (ESR), and fibrinogen—before and after the initiation of SGLT2i therapy.

## MATERIAL AND METHODS

### Patient population

In this retrospective, monocentric study, we analyzed patients diagnosed with heart failure admitted to Prof. Dr. Th. Burghele Clinical Hospital in Bucharest, Romania, between January 2021 and March 2023. The study included patients with a known diagnosis of chronic heart failure who were either admitted for acute decompensated heart failure or presented for scheduled follow-up evaluations. The exclusion criteria were age below 18 years, presence of systemic autoimmune or autoinflammatory diseases, advanced chronic kidney disease (estimated glomerular filtration rate [eGFR] < 10 mL/min/1.73 m^2^), and current use of medications with anti-inflammatory effects (non-steroidal or steroidal drugs, immunosuppressive agents, or biological drugs) for other comorbidities.

Patients were evaluated at two visits: baseline (T0) and after 6 months (T1). They underwent a comprehensive clinical assessment, laboratory testing, and echocardiography at each visit. The biological evaluation of the patients included a complete blood count, assessment of the three non-specific inflammatory markers (CRP, ESR, and fibrinogen), creatinine and blood urea nitrogen, electrolytes, glycemic profile, liver enzymes, iron and ferritin levels, urinalysis sediment and culture, and the intracardiac filling pressures marker amino-terminal pro-brain natriuretic peptide (NT-proBNP). All patients had their treatment regimen adjusted as needed to align with an optimally tolerated dose of guideline-directed medical treatment (OGMT) for heart failure, which included the initiation of SGLT2i.

The primary endpoint of the study was to evaluate if SGLT2i was able to reduce at least one of the inflammatory markers (CRP, ESR, fibrinogen). The secondary endpoint was to analyze whether the reduction in inflammatory markers from T0 to T1 differed among heart failure phenotypes (HFrEF, HFmrEF, and HFpEF).

### Statistical analysis

Patient data, including demographic, clinical, biological, and imaging information, were initially organized using Microsoft Excel (version 2404). Statistical analyses were performed with SPSS version 26 (SPSS Inc., Chicago, IL). Descriptive statistics were computed for the dependent variables (CRP, ESR, and fibrinogen). These three markers of inflammation were chosen to be evaluated because of their practical applicability (CRP, ESR, and fibrinogen are the most frequently used markers of inflammation by physicians in the hospital where this study was performed). In order to identify the change in time of each inflammation marker, a new variable was defined as the arithmetic difference between T1 and T0 values (a positive value indicates an increase and a negative value indicates a decrease). These different variables were analyzed using *t*-tests to compare patients who received SGLT2i versus those who did not, with Levene’s F test used to assess inter-group variance heterogeneity. Based on the significant *t*-tests obtained, repeated-measures analyses were conducted—a separate analysis for each of the three dependent variables of interest. A two-tailed significance level of 95% (*P* < 0.05) was considered for all statistical tests.

## RESULTS

### Descriptive statistics

A total of 220 patients meeting the inclusion and exclusion criteria were enrolled between January 2021 and March 2023. Among these patients, 111 were classified as having HFrEF, 23 as HFmrEF, and 86 as HFpEF. Demographic characteristics, comorbidities, and symptom profiles are summarized in Table 1.

**Table 1:** Descriptive statistics of the study group

	HFrEF *(*n* =* 111)	HFmrEF *(*n* =* 23)	HFpEF (*n* = 86)
**Comorbidities**			
BP (mean)			
Systolic	130.40 (90-230)	140.00 (100-180)	135.87 (80-190)
Diastolic	78.53 (50-134)	81.91 (60-100)	76.35 (50-105)
Chronic coronary syndrome	92 (82.9%)	11 (47.8%)	59 (68.6%)
Acute coronary syndrome	52 (46.8%)	4 (17.4%)	12 (14.0%)
Dilated cardiomyopathy	53 (47.7%)	3 (13.0%)	3 (3.5%)
Hypertrophic cardiomyopathy	3 (2.7%)	1 (4.3%)	12 (14.0%)
Restrictive cardiomyopathy	6 (5.4%)	--	1 (1.2%)
Atrial fibrillation	50 (45.0%)	17 (73.9%)	45 (52.3%)
Diabetes	51 (45.9%)	13 (56.5%)	32 (37.2%)
Dyslipidemia	96 (86.5%)	17 (73.9%)	79 (91.9%)
Cerebrovascular events	5 (4.5%)	--	10 (11.6%)
COPD	25 (22.5%)	3 (13.0%)	10 (11.6%)
CKD	77 (69.4%)	17 (73.9%)	47 (54.7%)
**Symptoms**			
Dyspnea (class)			
NYHA 1	3 (3.7%)	1 (4.3%)	8 (9.3%)
HYHA 2	34 (30.6%)	12 (52.2%)	54 (62.8%)
NYHA 3	58 (52.3%)	10 (43.5%)	21 (24.4%)
NYHA 4	16 (14.4%)	--	3 (3.5%)
Cough	26 (23.4%)	2 (8.7%)	14 (16.3%)
Asthenia/fatigability	92 (82.9%)	16 (69.6%)	65 (75.6%)
Angina	62 (55.9%)	7 (30.4%)	45 (52.3%)
Palpitations	88 (79.3%)	15 (65.2%)	64 (74.4%)
Syncope	7 (6.3%)	1 (4.3%)	11 (12.8%)

BP, Blood pressure; COPD, Chronic obstructive pulmonary disease; CKD, Chronic kidney disease; NYHA, New York Heart Association

The demographic data, comorbidities, and symptoms were recorded from the medical registries. In the overall cohort, 112 patients (50.9%) were men, with a mean age of 70.93 years. Within the subgroups, 64% of HFrEF patients, 65% of HFmrEF patients, and 29% of HFpEF patients were men. The mean age of patients was quite similar between the three categories: 68.2 years for HFrEF, 69.83 years for HFmrEF, and 74.62 years for HFpEF. Regarding body mass index (BMI), 72 patients (32%) were overweight, 54 (24%) had grade I obesity, 14 (6%) had grade II obesity, and only two (<1%) had grade III obesity. Also, 49 of the patients (32%) were smokers, while 27 (13%) had chronic alcohol consumption as a cardiovascular risk factor.

On physical examination, signs of systemic congestion were most prominent in the HFrEF subgroup: 37% had jugular distension, 55% exhibited peripheral edema, and 13% presented with anasarca. At the 6-month follow-up (T1), 123 patients attended a scheduled re-evaluation, while 84 patients were readmitted due to an episode of acute decompensated heart failure. Data distribution analysis (including skewness, kurtosis, and normality tests) indicated non-normality with possible outliers. After identifying a significant correlation between elevated inflammatory markers and markers of active urinary infection (e.g., leukocytosis and leukocyturia), 13 patients with inflammatory marker values above three standard deviations were excluded. The final analysis included 207 patients.

### Anti-inflammatory effect of SGLT2i on inflammatory biomarkers

Our research aimed to determine if patients with CHF receiving SGLT2i had a reduction in inflammatory markers compared to patients receiving OGMT according to the 2021 European Society of Cardiology (ESC) CHF guidelines without SGLT2i. We compared the dynamics of inflammatory markers—CRP, ESR, and fibrinogen—between T0 (baseline) and T1 (6-month follow-up) using a multivariate analysis of variance (MANOVA) across the three heart failure phenotypes (HFrEF, HFmrEF, and HFpEF). Overall, MANOVA did not reveal significant differences among the three phenotypes.

We then analyzed separately the patients who, at the second evaluation (T1), were admitted for acute decompensated heart failure versus those who came for a routine re-evaluation. In the subgroup without decompensation, we identified significant differences in the ESR dynamics between patients with HFrEF and HFmrEF. We also observed differences between patients with HFpEF and HFmrEF, with no difference in CRP or fibrinogen levels. Data on the multivariate analysis are presented in Table 2, and the post-hoc analysis based on estimated marginal means is presented in Table 3.

**Table 2:** MANOVA results for changes in inflammatory biomarkers (T1 – T0) among patients without decompensated CHF

		df	F	Sig.
CRP difference T1-T0	Between Groups	2	1.237	.294
	Within Groups	118		
	Total	120		
ESR difference T1-T0	Between Groups	2	4.149	.019
	Within Groups	89		
	Total	91		
Fibrinogen difference T1-T0	Between Groups	2	.048	.953
	Within Groups	58		
	Total	60		

**Table 3:** Post-hoc analysis of inflammatory biomarker changes (T1–T0) among patients without decompensated CHF stratified by heart failure phenotype

Dependent Variable	(I) Ejection Fraction	(J) Ejection Fraction	Mean Difference (I-J)	Sig.
CRP difference T1-T0	HFrEF	HFmrEF	-5.80584	.170
		HFpEF	-3.49567	.241
	HFmrEF	HFrEF	5.80584	.170
		HFpEF	2.31016	.587
	HFpEF	HFrEF	3.49567	.241
		HFmrEF	-2.31016	.587
ESR difference T1-T0	HFrEF	HFmrEF	-13.49444*	.005
		HFpEF	-3.14973	.370
	HFmrEF	HFrEF	13.49444*	.005
		HFpEF	10.34472*	.028
	HFpEF	HFrEF	3.14973	.370
		HFmrEF	-10.34472*	.028
Fibrinogen difference T1-T0	HFrEF	HFmrEF	-12.24242	.783
		HFpEF	-5.63636	.838
	HFmrEF	HFrEF	12.24242	.783
		HFpEF	6.60606	.886
	HFpEF	HFrEF	5.63636	.838
		HFmrEF	-6.60606	.886

The differences between the mean values of inflammatory markers are listed in Table 4. For the HFrEF phenotype, ESR values were similar at T0 and T1 (28.95 mm/h at T0 and 28.18 mm/h at T1). In contrast, for the HFmrEF category, ESR values increased from a mean value of 18.13 mm/h at T0 to a value of 27.25 mm/h at T1. In the HFpEF category, ESR values decreased between T0 and T1 from a mean value of 27.87 mm/h to 24.44 mm/h.

**Table 4:** Differences in mean values of inflammatory markers among patients without decompensated CHF (differences between HFrEF, HFmrEF, and HFpEF subgroups)

		*n*	Mean	Std. Deviation	95% Confidence Interval for Mean	
					Lower Bound	Upper Bound
ESR T0	HFrEF	44	28.95	19.641	22.98	34.93
	HFmrEF	15	18.13	17.225	8.59	27.67
	HFpEF	45	27.87	24.683	20.45	35.28
	Total	104	26.92	21.795	22.68	31.16
ESR T1	HFrEF	40	28.18	21.618	21.26	35.09
	HFmrEF	16	27.25	24.635	14.12	40.38
	HFpEF	45	24.44	19.984	18.44	30.45
	Total	101	26.37	21.263	22.17	30.56
CRP T0	HFrEF	54	12.6443	14.64639	8.6466	16.6420
	HFmrEF	17	5.7871	9.39972	.9542	10.6200
	HFpEF	50	6.1848	13.97833	2.2122	10.1574
	Total	121	9.0117	14.03752	6.4850	11.5383
CRP T1	HFrEF	54	7.3902	9.16056	4.8898	9.8905
	HFmrEF	17	6.3388	9.58829	1.4090	11.2687
	HFpEF	50	4.4264	5.34260	2.9080	5.9448
	Total	121	6.0178	7.93054	4.5903	7.4452
Fibrinogen T0	HFrEF	40	441.65	95.735	411.03	472.27
	HFmrEF	8	375.50	83.957	305.31	445.69
	HFpEF	30	395.97	103.047	357.49	434.44
	Total	78	417.29	99.744	394.81	439.78
Fibrinogen T1	HFrEF	40	433.98	111.012	398.47	469.48
	HFmrEF	11	382.82	58.179	343.73	421.90
	HFpEF	31	396.26	76.411	368.23	424.29
	Total	82	412.85	94.669	392.05	433.65

For patients who were admitted at T1 due to acute decompensated heart failure, MANOVA revealed significant differences in fibrinogen dynamics among all three subgroups (HFrEF vs. HFmrEF, HFrEF vs. HFpEF, and HFmrEF vs. HFpEF). No difference in mean values was identified in the case of CRP and ESR levels. Tables 5 and 6 show MANOVA and post-hoc analysis (based on estimated marginal means).

**Table 5:** Differences in inflammatory biomarkers between T1 and T0 among patients with decompensated CHF (differences between HFrEF, HFmrEF, and HFpEF subgroups) MANOVA

		df	F	Sig.
CRP difference T1-T0	Between Groups	2	.095	.910
	Within Groups	73		
	Total	75		
ESR difference T1-T0	Between Groups	2	.614	.545
	Within Groups	54		
	Total	56		
Fibrinogen difference T1-T0	Between Groups	2	5.090	.014
	Within Groups	25		
	Total	27		

**Table 6:** Post-hoc analysis of changes in inflammatory biomarkers (T1–T0) among patients with decompensated CHF, stratified by heart failure phenotype (HFrEF, HFmrEF, and HFpEF)

Dependent Variable	(I) Ejection Fraction	(J) Ejection Fraction	Mean Difference (I-J)	Std. Error
CRP difference T1-T0	HFrEF	HFmrEF	2.01436	6.97893
		HFpEF	-.94263	3.82936
	HFmrEF	HFrEF	-2.01436	6.97893
		HFpEF	-2.95699	7.09798
	HFpEF	HFrEF	.94263	3.82936
		HFmrEF	2.95699	7.09798
ESR difference T1-T0	HFrEF	HFmrEF	11.01379	9.93903
		HFpEF	1.76162	5.73096
	HFmrEF	HFrEF	-11.01379	9.93903
		HFpEF	-9.25217	10.12788
	HFpEF	HFrEF	-1.76162	5.73096
		HFmrEF	9.25217	10.12788
Fibrinogen	HFrEF	HFmrEF	161.82456*	55.57753
difference T1-T0		HFpEF	-31.67544	41.89314
	HFmrEF	HFrEF	-161.82456*	55.57753
		HFpEF	-193.50000*	63.25726
	HFpEF	HFrEF	31.67544	41.89314
		HFmrEF	193.50000*	63.25726

The mean values for fibrinogen increased in the HFrEF subgroup from 369 mg/dl up to 403 mg/dl, decreased in the HFmrEF subgroup from 492 mg/dl to 364 mg/dl, and increased for the HFpEF subgroup from 400 mg/dl to 444 mg/dl. Values are presented in Table 7.

**Table 7:** Differences in mean values of inflammatory markers among patients with decompensated CHF (differences between HFrEF, HF-mrEF, and HFpEF subgroups)

		*n*	Mean	Std. Deviation	95% Confidence Interval for Mean	
					Lower Bound	Upper Bound
Fibrinogen T0	HFrEF	25	369.96	71.322	340.52	399.40
	HFmrEF	3	492.33	95.296	255.60	729.06
	HFpEF	14	400.64	116.562	333.34	467.94
	Total	42	388.93	93.687	359.73	418.12
Fibrinogen T1	HFrEF	31	403.77	89.154	371.07	436.48
	HFmrEF	5	364.00	49.168	302.95	425.05
	HFpEF	17	444.59	103.623	391.31	497.87
	Total	53	413.11	93.184	387.43	438.80
CRP T0	HFrEF	39	10.3664	14.70876	5.5984	15.1344
	HFmrEF	6	4.2383	2.48318	1.6324	6.8443
	HFpEF	31	6.2187	7.42016	3.4970	8.9404
	Total	76	8.1908	11.72046	5.5125	10.8690
CRP T1	HFrEF	39	12.5241	14.13259	7.9428	17.1054
	HFmrEF	6	4.3817	2.87296	1.3667	7.3967
	HFpEF	31	9.3190	13.98601	4.1889	14.4491
	Total	76	10.5739	13.62668	7.4601	13.6878
VSH T0	HFrEF	34	20.68	13.904	15.83	25.53
	HFmrEF	5	30.40	19.269	6.47	54.33
	HFpEF	25	26.36	21.373	17.54	35.18
	Total	64	23.66	17.612	19.26	28.06
VSH T1	HFrEF	33	24.64	17.902	18.29	30.98
	HFmrEF	5	21.80	15.073	3.08	40.52
	HFpEF	27	26.37	17.696	19.37	33.37
	Total	65	25.14	17.416	20.82	29.45

Because of the differences in ESR and fibrinogen levels, in the next step, we performed chi-square tests and repeated measures tests to see if the difference was correlated with the initiation of SGLT2i. The dependent variables for test-retest analysis were inflammatory markers, while the between-subjects parameters were HF phenotypes. The result was significant only for fibrinogen dynamics (*P* = 0.017). The values for the SGLT2i test-retest with each dependent variable (fibrinogen, ESR, and CRP) are presented in Table 8.

**Table 8:** Test-retest regarding the interaction between SGLT2i and inflammatory markers (fibrinogen, ESR, and CRP) among all patients

Effect	Value	F	Hypothesis df	Error df	Sig.	Partial Eta Squared
Fibrinogen T0-T1 * EF * SGLT2i	.895	4.289	2.000	73.000	.017	.105
ESR T0-T1 * EF * SGLT2i	.992	.373	3.000	131.000	.773	.008
CRP T0-T1 * EF * SGLT2i	.998	.132	3.000	179.000	.941	.002

**Figure 1:**
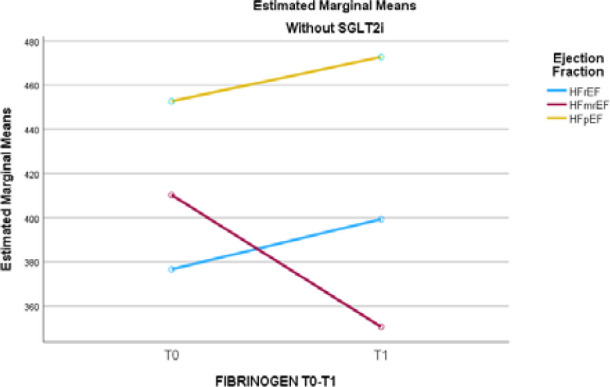
Estimated marginal means for fibrinogen across T0 and T1 across HFrEF, HFmrEF, and HFpEF phenotypes in patients without SGLT2i

**Figure 2:**
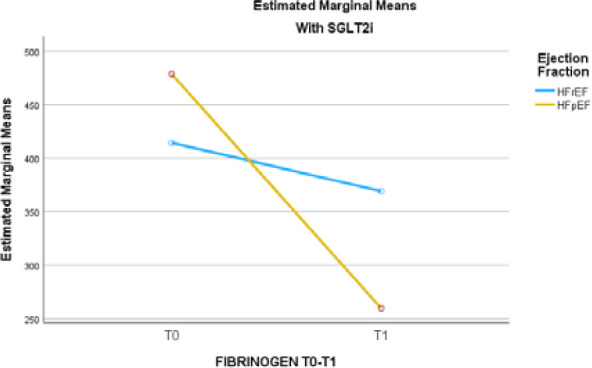
Estimated marginal means for fibrinogen at T0 and T1 in HFrEF and HFpEF patients receiving SGLT2i

Based on the estimated mean values, patients receiving SGLT2i had a reduction in fibrinogen levels between T0 and T1 in both the HFrEF and HFpEF phenotypes. In contrast, patients who did not receive SGLT2i showed increased fibrinogen levels during the same interval in the HFrEF and HFpEF subgroups. The number of patients receiving SGLT2i from the HFmrEF subgroup was too small to attain a statistical test-retest result. The mean values are presented in Table 9, and the trends are displayed in Figures 1 and 2.

**Table 9:** Mean values of fibrinogen at each evaluation (T0 and T1) for each phenotype of HF (HFrEF, HFmrEF, HFpEF)

EF	SGLT2i T0	Fibrinogen	Mean	Std.Error	95% Confidence Interval	
					Lower Bound	Upper Bound
HFrEF	NO	T0	376.658	48.047	280.832	472.484
		T1	399.336	46.096	307.401	491.271
	YES	T0	414.330	56.856	300.935	527.725
		T1	369.146	54.547	260.356	477.937
HFmrEF	NO	T0	410.318	53.274	304.067	516.570
		T1	350.636	51.111	248.699	452.573
	YES	T0				
		T1				
HFpEF	NO	T0	452.723	64.511	324.060	581.386
		T1	472.703	61.891	349.265	596.142
	YES	T0	478.557	129.246	220.785	736.330
		T1	259.759	123.998	12.453	507.065

## DISCUSSION

Our study focused on investigating the possible influence of SGLT2i on the level of inflammatory biomarkers (CRP, ESR, and fibrinogen) from the moment of initiation (T0) to follow-up (T1). In the general evaluation, no significant differences were identified regarding CRP, ESR, or fibrinogen trends across the three HF phenotypes (HFrEF, HFmrEF, or HFpEF). However, when analyzed separately, a difference in ESR trend was observed between HFrEF and HFmrEF patients. For the HFrEF phenotype, ESR values were similar at T0 and T1 (mean value at T0 – 28.95 mm/h and mean value at T1 – 28.18 mm/h), while for the HFmrEF, ESR values increased from a mean value of 18.13 mm/h at T0 to a value of 27.25 mm/h at T1. There was no difference in the CRP and fibrinogen trends (from T0 to T1) between HF phenotypes.

In the statistical analysis of patients admitted at T1 for acute decompensation of HF, the mean values of fibrinogen increased in the HFrEF subgroup from 369 mg/dl to 403 mg/dl. A similar trend was observed in the HFpEF subgroup, where the fibrinogen mean values increased from 400 mg/dl to 444 mg/dl, but not in the HFmrEF subgroup, where the values decreased from 492 mg/dl to 364 mg/dl. For this category of patients with HF decompensation, there was no significant difference in CRP or ESR dynamics between T0 and T1 across the HF phenotypes.

The second phase of our study included a detailed comparison of SGLT2i effects across HF phenotypes. Although MANOVA indicated significant differences in ESR (for stable patients) and fibrinogen (for decompensated patients), subsequent chi-square and repeated-measures analyses revealed statistical significance only for the interaction between SGLT2i and fibrinogen levels (*P* = 0.017). In the case of ESR, the *P* value was 0.773, and in the case of CRP, the *P* was 0.941. This means the result was not even marginally significant, supporting the conclusion that SGLT2i only influenced fibrinogen dynamics.

Based on the estimated mean values, patients who did not receive SGLT2i after T0 experienced increased fibrinogen levels between T0 and T1. In the case of HFrEF patients, the increase was from a mean fibrinogen level of 376 mg/dl to 399 mg/dl. For patients classified as HFpEF, fibrinogen mean values increased from 452 mg/dl to 472 mg/dl. However, HFmrEF patients who also did not receive SGLT2i and were treated with the other anti-remodeling medication classes had a decrease in fibrinogen levels from 410 mg/dl to 350 mg/dl.

The test-retest performed on patients receiving SGLT2i showed a significant decrease in fibrinogen levels between T0 and T1, both in HFrEF and HFpEF phenotypes. In the HFrEF subgroup, the mean fibrinogen levels decreased from 414 mg/dl to 369 mg/dl. The greatest decrease in fibrinogen level was recorded in HFpEF patients who received SGLT2i, from 478 mg/dl to 259 mg/dl.The number of patients receiving SGLT2i from the HFmrEF subgroup was too small to obtain a statistical result in test-retests. These results seem to contradict the data from Theofilis *et al*. study, which showed that administration of SGLT2i l lowered levels of CRP, TNF-a, and IL-6; however, it is important to note that their study was conducted on animal subjects [17].

Also, a meta-analysis conducted by Wang *et al*. previously showed that SGLT2i decreased the levels of CRP, ferritin, and leptin of patients with type 2 diabetes mellitus, when compared to placebo or standard antidiabetic medication [18].

Recent studies also showed that SGLT2i had anti-inflammatory effects in patients with type 2 diabetes mellitus, reducing not only CRP levels but also TNF-α and IL-6 [19]. However, few studies have evaluated the effect of SGLT2i on fibrinogen and ESR levels. One recent article disproved the theory that SGLT2i reduces the inflammatory marker levels, showing no significant effect on CRP, ESR, or fibrinogen levels after 6 months of treatment in a group of patients with CHF [20]. Most efforts focused on measuring the effect of SGLT2i on the levels of pro-inflammatory cytokines, such as IL-1, IL-6, and TNF-α [21].

### Limitations

One of the most important limitations was the fact that our study was a retrospective, observational study. At the same time, the number of patients who did receive SGLT2i was not equal to the number of patients not receiving this class of medication. Another limitation is the high proportion of patients who presented at T1 with acutely decompensated HF (84 out of 207), which may have altered the levels of inflammatory markers. Furthermore, associated comorbidities could have positively influenced (i.e., increased) the levels of biomarkers, introducing a confounding factor into the final analysis. Lastly, many patients with HFmrEF and HFpEF were not assigned SGLT2i treatment after T0 because, at the beginning of enrollment in 2021, the ESC Guideline for HF treatment recommended SGLT2i only for HFrEF patients (first-class recommendation with A level of evidence). The recommendation to initiate SGLT2i across the whole spectrum of EF was introduced only in the Focused Update of the ESC HF Guideline in 2023.

## CONCLUSION

In conclusion, SGLT2i is the first class of anti-remodeling medication for heart failure that is mandatory, irrespective of LVEF. The cardiovascular benefits of SGLT2i in the treatment of HF seem to encompass many pharmacological mechanisms. One of the explanations behind this effect is the anti-inflammatory properties of SGLT2i. In addition to improving glycemic control, SGLT2i inhibits the renin-angiotensin-aldosterone pathway of neurohormonal activation —a system that induces peripheral vascular stiffness with shear stress on endothelial structure. At the same time, SGLT2i stops the process of cardiac remodeling and the oxidative stress that damages the cardiomyocytes by inhibiting both neurohormonal systems.

Our study showed that patients with HFrEF and HFpEF who did not receive SGLT2i, as part of their HF treatment, had increased fibrinogen levels between T1 and T0, while the CRP and ESR remained the same between the two visits. On the other hand, patients with HFrEF and HFpEF for whom SGLT2i was initiated at T0 had a statistically significant decrease in fibrinogen levels (*P* = 0.017), the greatest reduction observed in patients with the HFpEF phenotype. In the subgroup of patients with HFmrEF, no difference was observed in fibrinogen, CRP, or ESR dynamics, either with or without SGLT2i. New studies with larger cohorts of patients are required to gather more evidence in decoding the anti-inflammatory effects of SGLT2i across the whole spectrum of patients with HF.

## Data Availability

The original contributions presented in the study are included in the article. Further inquiries can be directed to the corresponding author.
